# Discovering Genes Involved in Alcohol Dependence and Other Alcohol Responses: Role of Animal Models

**DOI:** 10.35946/arcr.v34.3.13

**Published:** 2012

**Authors:** Kari J. Buck, Lauren C. Milner, Deaunne L. Denmark, Seth G.N. Grant, Laura B. Kozell

**Affiliations:** **Kari J. Buck, Ph.D.,***is a professor of behavioral neuroscience at the Oregon Health & Science University and a research scientist at the Department of Veterans Affairs Medical Center, Portland, Oregon.*; **Lauren C. Milner, Ph.D.,***is a postdoctoral fellow at Stanford University, Palo Alto, California.*; **Deaunne L. Denmark, M.D., Ph.D.,***senior research associates, the Portland Alcohol Research Center and Department of Behavioral Neuroscience, Department of Veterans Affairs Medical Center and Oregon Health & Science University, Portland, Oregon.*; **Seth G.N. Grant, Ph.D.,***is a professor of molecular neuroscience at the Wellcome Trust Sanger Institute, Cambridge, United Kingdom.*; **Laura B. Kozell, Ph.D.,***are senior research associates, the Portland Alcohol Research Center and Department of Behavioral Neuroscience, Department of Veterans Affairs Medical Center and Oregon Health & Science University, Portland, Oregon.*

**Keywords:** Alcoholism, alcohol dependence, alcohol withdrawal, risk factors, genetic factors, genetic theory of AODU, genetic trait, DNA, QTL mapping, quantitative trait loci (QTLs), quantitative trait genes (QTGs), animal models

## Abstract

The genetic determinants of alcoholism still are largely unknown, hindering effective treatment and prevention. Systematic approaches to gene discovery are critical if novel genes and mechanisms involved in alcohol dependence are to be identified. Although no animal model can duplicate all aspects of alcoholism in humans, robust animal models for specific alcohol-related traits, including physiological alcohol dependence and associated withdrawal, have been invaluable resources. Using a variety of genetic animal models, the identification of regions of chromosomal DNA that contain a gene or genes which affect a complex phenotype (i.e., quantitative trait loci [QTLs]) has allowed unbiased searches for candidate genes. Several QTLs with large effects on alcohol withdrawal severity in mice have been detected, and fine mapping of these QTLs has placed them in small intervals on mouse chromosomes 1 and 4 (which correspond to certain regions on human chromosomes 1 and 9). Subsequent work led to the identification of underlying quantitative trait genes (QTGs) (e.g., *Mpdz*) and high-quality QTG candidates (e.g., *Kcnj9* and genes involved in mitochondrial respiration and oxidative stress) and their plausible mechanisms of action. Human association studies provide supporting evidence that these QTLs and QTGs may be directly relevant to alcohol risk factors in clinical populations.

A host of biological (i.e., genetic) and environmental factors interact throughout the addictive process to influence alcohol use and abuse. These processes are accompanied by a number of behavioral and neural events that include, but are not limited to, changes in the motivational effects of ethanol (both rewarding and aversive), tolerance to some effects of ethanol, and withdrawal when ethanol use is discontinued (Koob and Le Moal 2008). The role of genetics in individual differences in the degree and/or the rate of development of all such changes clearly has been illustrated in human genetic studies as well as studies using genetic animal models (for reviews, see Crabbe 2008 and references therein; Heath et al. 2011 and references therein). Although a few genes consistently have demonstrated a role in alcoholism or associated characteristics (i.e., subclinical markers known as endophenotypes) in human studies, their identification has relied heavily on prior knowledge of the physiological responses to alcohol. Thus, these genes primarily encode alcohol-metabolizing enzyme isoforms and neurotransmitter receptor subunits, both of which already had been known to be affected by alcohol (for more information, see the articles by Hurley and Edenberg, pp. 339–344, and by Borghese and Harris, pp. 345–354, in this issue). Moreover, the genetic variants identified to date do not wholly explain the complex genetic susceptibility to alcoholism. Accordingly, researchers need unbiased, systematic approaches to gene discovery in order to discover novel genes and mechanisms and translate them into improved treatment and prevention approaches. One promising approach to achieving this is to conduct genome-wide association studies (GWASs). However, human GWASs require large sample sizes to identify alcoholism susceptibility genes, and the studies published to date have been under-powered and show limited replicability (for a review, see Treutlein and Reitschel 2011 and references therein).

The use of preclinical (i.e., animal) models that closely approximate the clinical situation has been essential for elucidating genetic factors involved in the response to alcohol. Although no animal model can exactly duplicate alcoholism in humans, robust animal models for specific alcohol-related traits are useful for identifying potential determinants of liability in humans. These models include, but certainly are not limited to, the following:

- Animal models of ethanol self-administration, given that excessive ethanol consumption is a hallmark of alcohol use disorders in humans;
- Models of conditioned place preference and conditioned taste aversion to assess the motivational (i.e., rewarding and aversive) effects of ethanol;
- Models of ethanol sensitivity, because evidence from human studies indicates that sensitivity to the intoxicating effects of ethanol is a marker of genetic susceptibility to develop alcohol dependence; and
- Models of withdrawal, because physiological dependence on alcohol and associated withdrawal symptoms are thought to constitute a motivational force that perpetuates alcohol use and abuse.

For recent publications that address potential consilience between human alcohol dependence and animal models in more depth, the reader is referred to Crabbe and colleagues (2010), Ehlers and colleagues (2010), and Sher and colleagues (2010).

Studies using robust animal, and particularly mouse, models have been fundamental to unbiased searches for genetic determinants of ethanol responses. For example, researchers have used such models to detect and map quantitative trait loci (QTLs)—chromosomal regions containing or linked to the genes that underlie a quantitative, complex trait. These approaches have identified significant and suggestive QTLs for ethanol sensitivity (e.g., Bennett et al. 2006; Downing et al. 2006; Palmer et al. 2006), consumption (e.g., Belknap and Atkins 2001; Boyle and Gill 2008; Hitzemann et al. 2009; Phillips et al. 1998, 2010;Tarantino et al. 1998), withdrawal (Buck et al. 1997, 2002), conditioned aversion (Risinger and Cunningham 1998), conditioned place preference (Cunningham 1995), and tolerance (e.g., Bennett et al. 2007; Crabbe et al. 1994; Drews et al. 2010; Kirstein et al. 2002). The identification of specific genes (i.e., quantitative trait genes [QTGs], which carry allelic variations in the DNA that affect their expression and/or the structure of the product that they code for) that underlie QTL phenotypic effects and elucidation of their mechanisms of action is a crucial next step in the translation of such preclinical research.

Successful strategies to identify genes involved in alcohol dependence and other alcohol-related responses most often have relied upon evidence from several sources (e.g., Shirley et al. 2004). This article summarizes the detection, confirmation, and fine mapping of several QTLs that have large effects on ethanol withdrawal in mice using a variety of approaches, including robust behavioral models of physiological dependence and associated withdrawal following acute and chronic ethanol exposure, positional cloning, sequence and expression analyses, and novel genetic animal models. In addition, the article discusses progress toward the identification of the underlying QTGs, their mechanisms of action, and their potential broader effects on behavioral responses to ethanol.

## QTLs and QTGs Associated With Alcohol Dependence and Other Alcohol Effects

### The Mpdz Gene

Using a robust behavioral model of physiological dependence, Buck and colleagues (1997) identified a QTL on chromosome 4 that accounts for up to 26 percent of the genetic variance in withdrawal-associated convulsions following acute and chronic ethanol exposure in mice. Positional cloning using novel interval-specific congenic mouse strains^1^ narrowed this QTL to a 1.8-Mb interval with conserved colocalization of genes (i.e., shared synteny) on human chromosome 9p23–p22.3.^2^ Detailed expression and sequence analyses subsequently identified a single QTG candidate, *Mpdz*, which encodes a protein called multi-PDZ domain protein (MPDZ/MUPP1) (Shirley et al. 2004). Standard inbred and congenic strain analyses suggested that lower *Mpdz* expression and/or certain variations in the amino acid sequence of the encoded MPDZ protein were associated with more severe ethanol withdrawal (Fehr et al. 2002, 2004; Shirley et al. 2004). However, direct evidence that *Mpdz* affects ethanol withdrawal behavior in the intact organism (i.e., in vivo) currently is lacking. Investigators now are addressing this issue in ongoing studies using novel animal models in which either a foreign *Mpdz* gene has been introduced (i.e., MPDZ transgenic [MPDZ-TG] mice) or in which *Mpdz* expression has been reduced (i.e., knockout [MPDZ-KO] mice). The MPDZ-TG animals show increased *Mpdz* expression compared with their wild-type (WT), non-TG littermates. Ongoing studies suggest that withdrawal-related hyperexcitability of the central nervous system (CNS), which can be assessed using handling-induced convulsions, may be less severe in MPDZ-TG mice than in WT littermates—in other words, the animals with increased MPDZ expression experience less severe withdrawal. Conversely, ongoing studies indicate that MPDZ-KO heterozygotes, which exhibit reduced MPDZ expression, may show more severe ethanol withdrawal than WT littermates. Thus, it seems that an inverse relationship exists between *Mpdz* expression and withdrawal severity. However, other studies have suggested that different variants of the *Mpdz* gene exist, resulting in variations in MPDZ protein structure that also may affect withdrawal severity (Fehr et al. 2002). Thus, it still is unclear whether one or both of these mechanisms underlie the effect of *Mpdz* on alcohol withdrawal. Nevertheless, because the strengths of the MPDZ-TG approach complement the limitations of the MPDZ-KO approach and vice versa, the finding that both approaches support a role for MPDZ in ethanol withdrawal is compelling.

Currently, little is known about how MPDZ function relates to alcohol dependence and withdrawal. PDZ-domain proteins regulate numerous aspects of the fate of proteins in the cell (e.g., targeting them to their appropriate location in the cell, stabilization in membranes, retention in a cell component called the endoplasmic reticulum, and endocytosis). MPDZ physically associates with certain proteins involved in brain signaling (i.e., neurotransmitter) systems. These include the following:

- Receptors for the neurotransmitter serotonin (5-HT) (e.g., the 5-HT_2C_ receptor) (Becamel et al. 2001);
- A receptor for the neurotransmitter γ-aminobutyric acid (GABA) (i.e., the GABA_B_ receptor) (Balasubramanian et al. 2007); and
- A protein called synaptic GTPase-activating protein (SynGAP). Through this association, MPDZ is involved in regulating the functions of the neurotransmitter glutamate, because after binding to SynGAP, MPDZ interacts (i.e., complexes) with one type of glutamate receptor (i.e., NR2B-containing NMDA receptors) to regulate the function of another type of glutamate receptor (i.e., synaptic AMPA receptors) (Krapivinsky et al. 2004).

MPDZ may affect withdrawal by altering the rate and/or fidelity of signal transduction mediated by one or more of the proteins with which it associates, particularly through its effects in a brain region(s) relevant to withdrawal. The striatum appears to be one such region, as ethanol-withdrawal–associated activation of this brain region is related to MPDZ status (Chen and Buck 2010). Within the striatum, virtually all neurons activated in alcohol-withdrawn mice produce both MPDZ and the 5-HT_2C_ receptor, and 30 percent of neurons also produce SynGAP (Chen and Buck 2010). Analyses of inbred mouse strains have indicated that MPDZ status is genetically correlated with convulsions induced by certain chemicals, with some of the strongest genetic correlations observed for chemicals affecting signaling pathways involving glutamate (Fehr et al. 2004). For example, two mouse strains known as C57BL/6J and DBA/2J differ both in the MPDZ variant they carry, affecting both MPDZ expression and structure (Shirley et al. 2004; Fehr et al. 2002), and markedly in handling-induced convulsions in response to agents that modify glutamate signaling (Fehr et al. 2004). Further, MPDZ congenic mice, which possess the QTL interval containing *Mpdz* from the C57BL/6J strain in a genetic background that is more than 99 percent DBA/2J DNA, demonstrate less severe ethanol withdrawal (Fehr et al. 2002) and less severe handling-induced convulsions than DBA/2J mice in response to a 5-HT_2C_ receptor blocker (i.e., SB242084) and a drug that activates GABA_B_ receptors (i.e., baclofen), but not in response to a GABA_A_ receptor channel blocker (Reilly et al. 2008). These findings indicate that MPDZ does not regulate seizure susceptibility in general and suggest that MPDZ may affect ethanol-withdrawal–associated CNS hyperexcitability through its effects on glutamate, 5-HT_2C_, and/or GABA_B_ receptor function. Ongoing neurophysiological studies using MPDZ genetic models can address this issue to provide mechanistic information.

### The Kcnj9 Gene

Buck and colleagues (1997, 2002) also identified a QTL on mouse chromosome 1 that accounts for 26 percent of the genetic variance in ethanol withdrawal convulsions in mice. Positional cloning using interval-specific congenic strains narrowed this QTL to a 0.44-Mb interval syntenic with human chromosome 1q23.2 (Kozell et al. 2009). This chromosome region contains a gene called *Kcnj9* which may be the QTG underlying this QTL. DBA/2J and chromosome 1 congenic mice (which possess a small QTL interval containing *Kcnj9* from the DBA/2J strain in a genetic background that is more than 99 percent C57BL/6J DNA) exhibit significantly more severe withdrawal from ethanol and other sedative drugs of abuse than C57BL/6J mice. Further, *Kcnj9* expression in the brain is significantly greater in chromosome 1 congenic and DBA/2J mice compared with C57BL/6J mice (Kozell et al. 2009). Additionally, mice in which the *Kcnj9* gene has been knocked out demonstrate less severe withdrawal than their WT littermates (Kozell et al. 2009). *Kcnj9* encodes a protein called GIRK3, which forms a subunit of a family of ion channels (i.e., G-protein–dependent inwardly-rectifying K+ channels) that are gated by G-proteins. GIRK channels primarily mediate the inhibitory effects of certain G-protein–coupled neurotransmitter receptors (Luscher et al. 1997).

Currently, little is known about GIRK3 function in the brain and the mechanism by which it can affect withdrawal. GIRK3 is widely expressed in the brain and forms ion channels together with the related GIRK2 protein subunit (Koyrakh et al. 2005; Labouebe et al. 2007; Torrecilla et al. 2002). One plausible mechanism by which GIRK3 may influence withdrawal is via its role in GABA_B_ receptor signaling. GABA_B_ receptor activation usually suppresses withdrawal symptoms in ethanol-dependent rats (Colombo et al. 2000; Knapp et al. 2007) and humans (Addolorato et al. 2006), and GIRK3-KO mice show increased GIRK-GABA_B_ receptor coupling efficiency in the brain compared with WT mice (Labouebe et al. 2007). Further, ethanol enhances GABA_B_ receptor mediated inhibitory transmission in brain neurons by facilitating GIRK currents (Federici et al. 2009; McDaid et al. 2008) and enhances GIRK currents evoked by GABA_B_ receptor activation (Kobayashi et al. 1999; Lewohl et al. 1999). In addition to altered GABA_B_ receptor function, GIRK3-KO mice have blunted behavioral responses to drugs that act at other G_i/o_-coupled receptors, including μ-opioid (Marker et al. 2002; but see Smith et al. 2008), cannabinoid and α_2_-adrenergic receptors (Smith et al. 2008). This suggests the potential involvement of GABA_B_ and additional G-coupled receptors in mediating GIRK3 effects on behavior. Of course, it should be kept in mind that deletion of a specific gene (in this case the *Kcnj9* gene) in KO animals may lead to developmental changes in other systems to compensate for this loss, and these compensatory changes can confound the interpretation of knockout studies. Future studies will be needed to assess GIRK coupling to GABA_B_ and similar receptors and its role in ethanol physiological dependence and withdrawal.

### QTLs and QTGs Related to Mitochondrial Respiration and Oxidative Stress

Additional studies using the interval-specific congenic mice carrying short segments of DBA/2J DNA in a background of C57BL/6J DNA detected, confirmed, and finely mapped a second QTL on mouse chromosome 1 with large effects on the predisposition to withdrawal following chronic and acute alcohol exposure (Kozell et al. 2008). This QTL maps to a 1.1-Mb interval syntenic with human 1q23.2–23.3. Although considerable evidence indicates that some genetic factors influence vulnerability to withdrawal from a variety of sedative drugs, this QTL does not influence pentobarbital withdrawal (Kozell et al. 2009) and provides a crucial clue as to the identity of the underlying QTG(s).

Detailed molecular analyses of this QTL interval have shown that it harbors 17 genes that exhibit genotype-dependent transcript expression between chromosome 1 congenic and C57BL/6J background strain mice and/or nonsynonymous sequence variation that changes the structure of the protein coded for by the gene, either one or both of which may underlie the QTL’s influence (Denmark and Buck 2008). Three of these genes (called *Sdhc*, *Ndufs2*, and *Ppox*) encode proteins found in cell organelles called mitochondria. Mitochondria supply most cellular energy and also are involved in pathways that help the cells avoid or deal with oxidative stress. This is notable because studies both in cultured cells (i.e., in vitro) and in vivo found that ethanol exposure introduces intense oxidative stress, largely through its effects on the mitochondria (Bailey 2003; Sun and Sun 2001).^3^ The protein products of *Sdhc* and *Ndufs2* are integral subunits of certain components (i.e., respiratory complexes I and II) involved in a series of biochemical reactions known as the mitochondrial electron transport chain. The protein encoded by *Ppox* catalyzes the penultimate step in the biosynthesis of heme, which is required for the function of cytochrome molecules that play a central role in mitochondrial respiratory function. Consistent with the hypothesis that these mitochondrial proteins could be related to ethanol withdrawal, studies found that mutations in the human genes *NDUFS2*^4^ (Ugalde et al. 2004) and *PPOX* (Gonzalez-Arriaza and Bostwick 2003) cause several inherited diseases that include seizures as a prominent symptom. Similar mutations in the gene corresponding to *Ndufs2* in the roundworm *Chromatia elegans* (which is called *gas-1*) creates ethanol hypersensitivity and increased oxidative stress (Kayser et al. 2001, 2003; Morgan and Sedensky 1995). Evidence from rat models suggests that ethanol withdrawal is accompanied by increases in oxygen-containing molecules that increase oxidative stress (i.e., reactive oxygen species) and that these increases correlate with withdrawal severity (Dahchour et al. 2005; Vallett et al. 1997).

Because the DBA/2J and C57BL/6J mice that were used to generate the interval-specific congenic strains differ significantly in the brain levels of some oxidative stress markers (Rebrin et al. 2007), this model system is highly attractive for conducting subsequent QTG and mechanistic analyses. Ongoing studies to address these issues suggest that a gene(s) within this QTL interval may affect mitochondrial respiratory complex structure in the brain and that antioxidant administration may reduce ethanol withdrawal severity in mice.

## Potential Roles of MPDZ and GIRK3 in Additional Responses to Ethanol

Alcohol consumption and preference show significant genetic correlation with ethanol withdrawal convulsion severity in independently tested groups of mice (Metten et al. 1998), suggesting that ethanol withdrawal and consumption/preference may share specific genetic contributions, which may include the *Mpdz* and *Kcnj9* genes and their encoded proteins. Consistent with this hypothesis, the same chromosomal region that contains *Kcnj9* also harbors QTLs for ethanol drinking (Tarantino et al. 1998) and for ethanol-conditioned aversion (Risinger and Cunningham 1998) and acute sensitivity to ethanol (Crabbe et al. 1994; Demarest et al. 1999). Moreover, analyses of standard and recombinant inbred animal strains have suggested that *Mpdz* status and/or expression may be genetically correlated with ethanol consumption and preference (behavioral data from Belknap et al. 1993; Fernandez et al. 1999; Gill et al. 1996; Rodriguez et al. 1994), ethanol-conditioned place preference and taste aversion (behavioral data from Broadbent et al. 2002; Cunningham 1995), and sensitivity and tolerance to ethanol-induced motor incoordination (behavioral data from Gallaher et al. 1996; Phillips et al. 1996; Rustay et al. 2003). These results suggest that *Mpdz* and *Kcnj9* may have roles in a wide variety of ethanol-related behaviors. The possibility that these QTGs play important roles in diverse responses to ethanol makes them important targets. Ongoing analyses using MPDZ and GIRK3 genetic models can begin to address their potential roles in ethanol consumption, preference, and other related behaviors.

## Human Relevance of QTGs Identified in Mice

**Figure 1:**
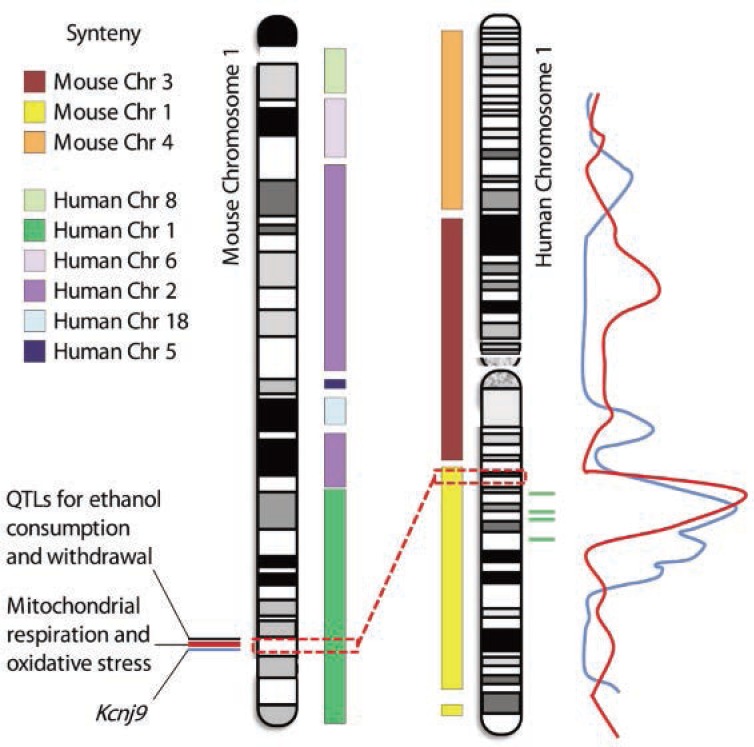
Potential synteny between mouse chromosome 1 and human chromosome 1 quantitative trait loci (QTLs). Human chromosome 1 shares primary conserved regions with mouse chromosomes 1, 3, and 4. Conversely, mouse chromosome 1 shares regions syntenic with human chromosomes 1, 2, 5, 6, 8, and 18. For both mouse and human chromosomes, additional smaller syntenic regions exist (not shown). Mouse chromosome 1 carries significant QTLs for physiological dependence and associated withdrawal following chronic and acute ethanol exposure, two of which have been finely mapped to small DNA intervals of 0.44 and 1.7 Mb (see blue and red lines next to mouse chromosome 1). High-quality quantitative trait gene (QTG) candidates have been identified within these two intervals, including *Kcnj9* and genes involved in mitochondrial respiration and/or oxidative stress. (Denmark and Buck 2009; Kozell et al. 2009). Another QTL for ethanol consumption and withdrawal has also been detected nearby (see black line) but has not yet been finely mapped. The dashed red boxes and line denote the two finely mapped mouse QTL intervals and the syntenic region of human chromosome 1 (1q23.2–1q23.3). Two human QTL studies have determined peak log of the odds of linkage (LOD) scores for alcoholism (red line; Hill et al. 2004) and for age of onset of drinking, harm avoidance, novelty seeking, and alcohol dependence (blue line; Dick et al. 2002) in this human chromosome 1 region. Four genetic markers (rs1229430, rs2001270, rs3753563, and rs84465) that are associated (P < 0.0001) with heaviness of drinking, alcohol use disorder, and/or alcohol dependence (Heath et al. 2011) are located in that same region (green lines). Thus, one or more human QTLs may be narrowed to a small syntenic interval of human chromosome 1 that harbors the homologs of high-quality QTG candidates identified in mice.

As reviewed here, studies in mouse models of various aspects of alcohol dependence have identified several QTLs with large effects on ethanol physiological dependence and associated withdrawal, have reduced these QTLs to small intervals of chromosomes 1 and 4 (which are syntenic to human chromosomes 1q23.2–23.3 and 9p23–p22.3, respectively), and have led to the description of a QTG or high-quality QTG candidate(s). Human association studies have provided evidence that the QTLs and QTGs identified in mice may be relevant to risk factors for alcoholism in clinical populations. For example, two studies have identified DNA regions associated with alcohol dependence on human chromosome 1q (LOD>3), and additional studies have provided supporting evidence for the association of 1q markers with alcoholism (for reviews, see Edenberg et al. 2010; Ehlers et al. 2010; Hansell et al. 2009; Heath et al. 2011; Joslyn et al. 2010) (see figure 1). These loci potentially are syntenic with the identified mouse chromosome 1 QTLs for alcohol consumption and withdrawal (Ehlers et al. 2010).

**Figure 2:**
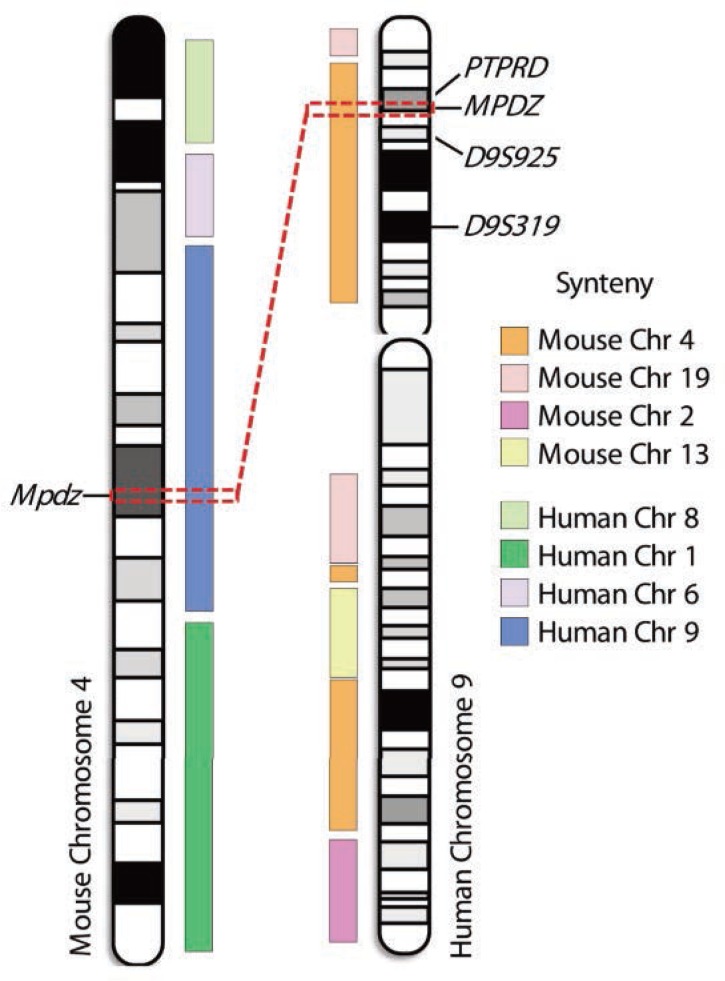
Potential synteny between mouse chromosome 4 and human chromosome 9 quantitative trait loci (QTLs). Human chromosome 9 shares primary conserved regions with mouse chromosomes 1, 4, 19, 2, and 13, and mouse chromosome 4 shares regions syntenic with human chromosomes 9, 8, 1, and 6. For both mouse and human chromosomes additional smaller syntenic regions exist (not shown). Mouse chromosome 4 carries a significant QTL for ethanol withdrawal that has been mapped to 1.8-Mb interval. Within this interval, a gene called *Mpdz* has been identified as a quantitative trait gene (QTG) candidate for ethanol withdrawal (Shirley et al. 2004). The dashed red boxes and line denote this 1.8-Mb QTL interval and syntenic region on human chromosome 9 (9p23–p22.3). A recent human association study for alcohol consumption (Tabakoff et al. 2010) found significant association with a DNA variation (i.e., single nucleotide polymorphism [SNP]) within the human gene *MPDZ* (P < 0.0001). Sequence variations in human *MPDZ* also may be associated with alcohol dependence (Karpyak et al. 2010). Thus, this gene has been implicated in studies using animal model and clinical populations. Additional markers near this region of human chromosome 9 may be associated with alcohol-related phenotypes, including age of onset of use (*D9S925*; Williams et al. 2005), predisposition to alcohol dependence (*D9S319*; Long et al. 1998), and alcohol response (*PTPRD*; Joslyn et al. 2010).

Several studies also have provided evidence for an association of markers on human chromosome 9p with alcoholism, but these associations only remain suggestive (Edenberg et al. 2010; Joslyn et al. 2010; Long et al. 1998; Williams et al. 2005). These markers potentially are syntenic with the mouse chromosome 4 QTL for which *Mpdz* has been proposed as a QTG (figure 2). In addition, limited human association studies using only small populations have implicated *MPDZ* as potentially involved in excessive alcohol consumption and risk for alcoholism (Karpyak et al. 2009; Tabakoff et al. 2009). Thus, this gene is a promising translational candidate for future work toward improving prevention and treatment of alcohol abuse in dependent individuals.

These findings suggest that QTL/QTG research in mice may help in the identification of new targets for improved therapeutic approaches and individualized strategies to treat and prevent dependence in humans. In some cases, a QTG can be the same in mice and humans, as has been found for some disorders (Mogil et al. 2003) and also may be the case for one or more of the examples above. In other cases, animal models may identify a relevant network operating in both species, within which many potential targets for pharmacologic interventions may exist. In either case, a comprehensive understanding of genetic variation, both in humans and informative animal models, is crucial to establishing its relationship to biological function (Collins et al. 2003). As more information becomes available, the mechanisms by which QTGs affect response to ethanol, and their potential role in alcohol dependence in humans, will become apparent.

## Footnotes

1 Congenic and interval-specific congenic strains refer to genetic animal models that are genetically identical to noncongenic (wild type) counterparts except for a limited genetic region (the congenic interval, which can range in size from moderately large to as small as 1 megabase). Through specific breeding strategies, a defined DNA segment from a donor strain’s genome is transferred to a different recipient strain. By analyzing a panel of interval-specific congenic strains, each with a different congenic interval, and determining which of the congenic strains “capture” the trait of interest (i.e., differ from noncongenic animals) and which do not, researchers can identify a small DNA segment that influences a trait of interest. This is an important step toward identifying the gene or genes within the DNA segment that affect the trait of interest.

2 When human chromosomes are prepared for microscopic examination, they take on an x-shaped form, with the shorter arm being labeled the p arm and the longer one the q arm. In addition, the chromosomes are stained with a special dye, giving them a characteristic pattern of light and dark bands that are numbered from the center of the chromosome outwards. Thus, a label such as 9p23 refers to a specific band on the short arm of chromosome 9.

3 In contrast, the sedative pentobarbital seems to have a neutral or anti-oxidative effect in the brain (Smith et al. 1980; Ueda et al. 2007).

4 By convention, gene names in animals are written in uppercase and lowercase and italicized. Gene names in humans are written in all caps and are italicized, whereas the acronyms for the encoded proteins are all caps but not italicized.
